# Identification of a 3-gene signature based on differentially expressed invasion genes related to cancer molecular subtypes to predict the prognosis of osteosarcoma patients

**DOI:** 10.1080/21655979.2021.1971919

**Published:** 2021-09-07

**Authors:** Yue Wan, Ning Qu, Yang Yang, Jing Ma, Zhe Li, Zhenyu Zhang

**Affiliations:** a Oncology Department, Jinzhou Central Hospital, Jin Zhou, Liao Ning, China; b Paediatrics, Jinzhou Central Hospital, Jinzhou, Liaoning, China; c Neurosurgery, Jinzhou Central Hospital, Jinzhou, Liaoning, China; d Nursing Department, Jinzhou Central Hospital, Jinzhou, Liaoning, China; e Hematology Department, First Affiliated Hospital of Jinzhou Medical University, Jinzhou, Liaoning, China; f Orthopedics Department, First Affiliated Hospital of Jinzhou Medical University, Jinzhou, Liaoning, China

**Keywords:** Osteosarcoma, mRNA, invasive gene signature, molecular subtype, prognosis

## Abstract

Invasion is a critical pathway leading to tumor metastasis. This study constructed an invasion-related polygenic signature to predict osteosarcoma prognosis. We initially determined two molecular subtypes of osteosarcoma, Cluster1 (C1) and Cluster2 (C2).. A 3 invasive-gene signature was established by univariate Cox analysis and least absolute shrinkage and selection operator (LASSO) Cox regression analysis of the differentially expressed genes (DEGs) between the two subtypes, and was validated in internal and two external data sets (GSE21257 and GSE39058). Patients were divided into high- and low-risk groups by their signature, and the prognosis of osteosarcoma patients in the high-risk group was poor. Based on the time-independent receiver operating characteristic (ROC) curve, the area under the curve (AUC) for 1-year and 2-year OS were higher than 0.75 in internal and external cohorts. This signature also showed a high accuracy and independence in predicting osteosarcoma prognosis and a higher AUC in predicting 1-year osteosarcoma survival than other four existing models. In a word, a 3 invasive gene-based signature was developed, showing a high performance in predicting osteosarcoma prognosis. This signature could facilitate clinical prognostic analysis of osteosarcoma.

## Introduction

As the most common malignant primary tumor in bone tissue, osteosarcoma, which mainly affects children and young people [1], often develops in the long bones such as femur, tibia, and humerus [2]. According to their characteristics and major stromal differentiation (osteoblastic, fibroblastic, chondroblastic, small-cell, telangiectatic high-grade surface, and extraskeletal), osteosarcoma can be divided into different subtypes [3]. At present, osteosarcoma is largely treated by preoperative and postoperative chemotherapy combined with surgical resection [4]. However, due to the strong invasiveness of osteosarcoma and its rapid progression, about 20% of osteosarcoma patients suffer from severe tumor metastasis at first diagnosis. More importantly, the prognosis of these patients remains unfavorable, with a long-term survival rate of only 20% to 30% [5,6]. Therefore, tumor metastasis is regarded as a main contributor leading to the poor prognosis of osteosarcoma.

As a process during which cancer cells spread from primary site to distal organ(s) [7], tumor metastasis consists of a series of complex cascade reactions interrelated through a series of adhesion interactions, invasive processes, and responses to chemotaxis stimuli [8]. Thus, invasion of cancer cells is regarded as one of the essential pathways resulting in tumor metastasis. In recent years, molecular signatures and markers of tumor metastasis have been increasingly reported. MengweiWu *et al.* developed a metastasis-related seven-gene signature based on RACGAP1, RARRES3, TPX2, MMP28, GPR87, KIF14, and TSPAN7 to predict the overall survival of pancreatic ductal adenocarcinoma (PDAC) patients. The seven genes were significantly related to the progression and overall survival of PDAC and have been regarded as potential targets for treatment [9]. JiahuaLiu *et al.* constructed a 6-gene signature prognostic hierarchical system (ITLN1, HOXD9, TSPAN11, GPRC5B, TIMP1, and CXCL13) based on colon cancer invasion-related genes with stable predictive efficiency in predicting the prognosis of patients [10]. Furthermore, another study also established a reliable and robust five-gene metastasis risk model (KRT8, MAFK, PTTG1, ENPP5, and INPP5J) based on the study of metastasis-related gene expression profiles to predict the prognosis of non-small cell lung cancer. At the same time, the five-gene metastasis signature was verified to be an independent prognostic factor [11]. Therefore, studying invasion-related markers has a great potential in improving the prognosis and survival rate of cancer patients. Various genomic analysis methods, including whole-genome and exome sequencing, transcriptome assessment of gene expression, and epigenetic modification, have been applied to analyze osteosarcoma samples, showing a heterogeneity of osteosarcoma researches [12]. Therefore, it could be argued that osteosarcoma subtypes should be classified according to invasion genes prior to the direct study of osteosarcoma invasion markers.

In this study, we developed the tumor subtypes of osteosarcoma based on metastasis-related genes, and screened three invasive gene markers related to the prognosis of osteosarcoma based on differentially expressed gene (DEGs) among tumor subtypes. Subsequently, a 3-gene signature based on invasive markers was constructed, assessed, and verified. The current findings improve the prognostic management of patients with metastatic osteosarcoma.

## Materials and methods

### Collection of clinical information and expression data of osteosarcoma

**Figure 1.**
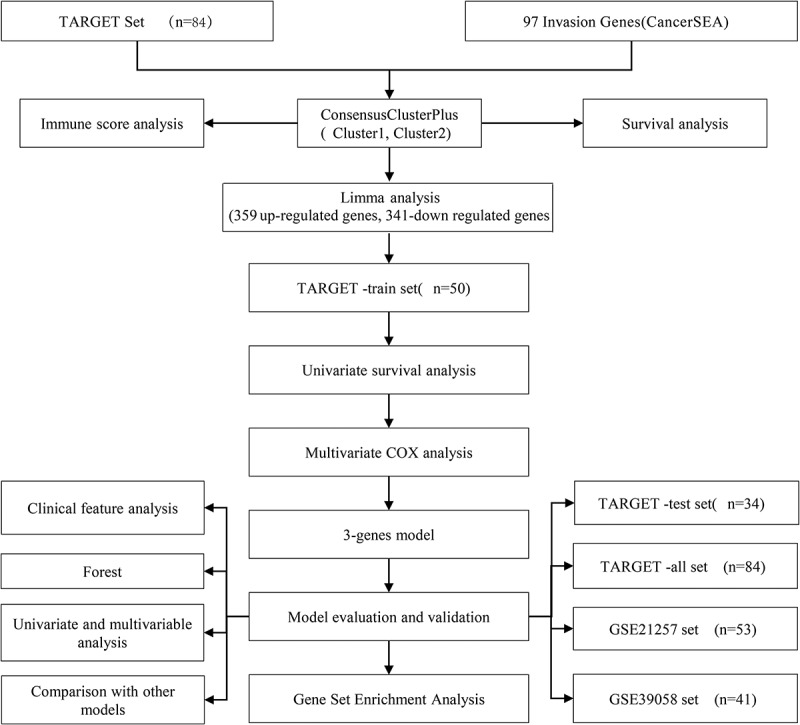
Flow chart of research and design

The Therapeutically Applicable Research to Generate Effective Treatments (TARGET) database (https://ocg.cancer.gov/programs/target) was used to download the RNA-Seq data and clinical follow-up information of osteosarcoma patients. After excluding the samples with incomplete clinical information, the expression profile information on 84 RNA-Seq samples was retained. The clinical data of GSE21257 and GSE39058 were from the Gene Expression Omnibus (GEO) database. Specifically, the GSE21257 data set contained 53 osteosarcoma samples with complete clinical information, and the data set GSE39058 contained 41 osteosarcoma samples with complete clinical information. For clinical data on the samples, see Table 1. A total of 97 invasion-related genes were obtained from the website of CancerSEA [13]. The whole process of the study is outlined in Figure 1.

**Table 1.** Sample clinical information for different data sets

Clinical Features	ARGET	GSE21257	GSE39058
Event			
0	55	30	29
1	29	23	12
Gender			
Male	47	34	21
Female	37	19	20
Age			
≤15	46	21	16
>15	38	32	25
Metastatic			
YES	21		
NO	63

### Consensus clustering by ConsensusClusterPlus

The invasion-related genes obtained in CancerSEA were filtered by TARGET to remove the genes that accounted for less than 50% of all the samples or with an expression level lower than 1. After filtering, the expression of invasive genes was extracted and subjected to univariate Cox analysis for screening invasion-related genes associated with osteosarcoma prognosis. Then, ConsensusClusterPlus [14](V1.48.0; Parameters: reps = 100, pItem = 0.8, pFeature = 1, distance = ‘spearman’) was employed to perform consistent clustering of the samples in the TARGET, according to the invasion-related genes associated with osteosarcoma prognosis. D2 and Euclidean distance were used as clustering algorithm and distance measure, respectively.

### Screening of DEGs and functional analysis

The differentially expressed genes between subgroups were analyzed using the Limma software package. The cutoff value was FDR < 0.05 and | fold change (FC) | > 1.5. The volcano map of DEGs and the heat map of DEGs were drawn using the R software package ggplot and pheatmap, respectively. The functional enrichment analysis of KEGG and GO of DEGs was carried out using WebGestaltR [15].

### Immune score estimation

The differences in stromal score, immune score, and ESTIMATE score of each subgroup samples were calculated using the R software package ESTIMATE [16]. MCP-counter [17] was applied to evaluate the score difference of 10 kinds of immune cells in the subgroup samples. The GSCA package (http://bioinfo.life.hust.edu.cn/web/GSCALite/) was used to evaluate the scores of 28 kinds of immune cells in osteosarcoma tissue samples. In addition, the enrichment scores of 22 immune cells in different subtype samples were calculated by CIBERSOTR [18].

### Construction of prognostic risk model

The samples in the TARGET data set were randomly grouped without putting back for 100 times, and the samples were divided into a training set (n = 50) and verification set (n = 34) at a ratio of 3:2. There was no significant difference in survival status, gender, age, or metastasis between the two groups (Table 2). Univariate Cox risk regression was performed for DEGs using the R package survival coxph function. Next, least absolute shrinkage and selection operator (LASSO) regression analysis was carried out to screen the genes related to osteosarcoma survival. The regression coefficients of these genes were calculated by multiple Cox regression analysis, and the risk score model was constructed according to gene expression level.

**Table 2.** TARGET training set and validation set sample information table

Clinical 1	TARGET-Train(n = 50)	TARGET-test(n = 34)	P-Value
Event			
0	35	20	0.4101
1	15	14	
Gender			
Male	31	16	0.2585
Female	19	18	
Age			
≤15	27	19	0.3353
>15	23	15	
Metastatic			
YES	14	7	0.6077
NO	36	27	

### Evaluation and validation of risk scoring model

Patients in each cohort were graded by their risk scores. The z score was calculated based on risk score, and patients with z score above 0 were in the high-risk group, while those with z score below 0 were in the low-risk group. The overall patient survival in the two groups were compared by Kaplan–Meier survival analysis. Receiver operating characteristic (ROC) curve, univariate, and multivariate Cox regression analysis was applied to evaluate the prognostic efficacy and independence of the risk score model. A nomogram was constructed by combining the independent prognostic factors obtained by multivariate Cox analysis, and its predictive accuracy was evaluated by drawing calibration curve. The net income of the model was calculated using the DCA diagram. In addition, patients were classified according to age, sex, and distant metastatic status to evaluate the correlation between risk groups based on prognostic signatures and clinical features.

### The risk scoring model was compared with other existing risk scoring systems

To highlight the advances of the risk scoring model developed in this study, we also compared the current risk scoring model with the previously constructed osteosarcoma scoring system [19–22]. According to the corresponding genes in these four models, the high- and low-risk groups of TARGET were divided into high- and low-risk groups using the same method. Survival curves and ROC curves of each model were drawn, and AUC values were compared.

## Results

This work aims to classify osteosarcoma subtypes and characterize its clinical characteristics based on invasion-related genes, and to construct a robust model to predict the prognosis of osteosarcoma. Two molecular subtypes of osteosarcoma were developed with metastasis-related genes. The OS and PFS of patients with C2 were found to be significantly longer than those of C1. We also identified three invasive gene markers related to the prognosis of osteosarcoma, according to the DEGs among tumor subtypes. A 3-gene signature based on invasive markers was constructed, and was introduced to divide patients into high- and low-risk groups. The results showed that the prognosis of osteosarcoma patients in the high-risk group was poor. This signature shows a high accuracy and independence in predicting osteosarcoma prognosis and a higher AUC in predicting 1-year osteosarcoma survival than the other four existing models.

### Consensus clustering identified two molecular subtypes of osteosarcoma invasion

**Figure 2.**
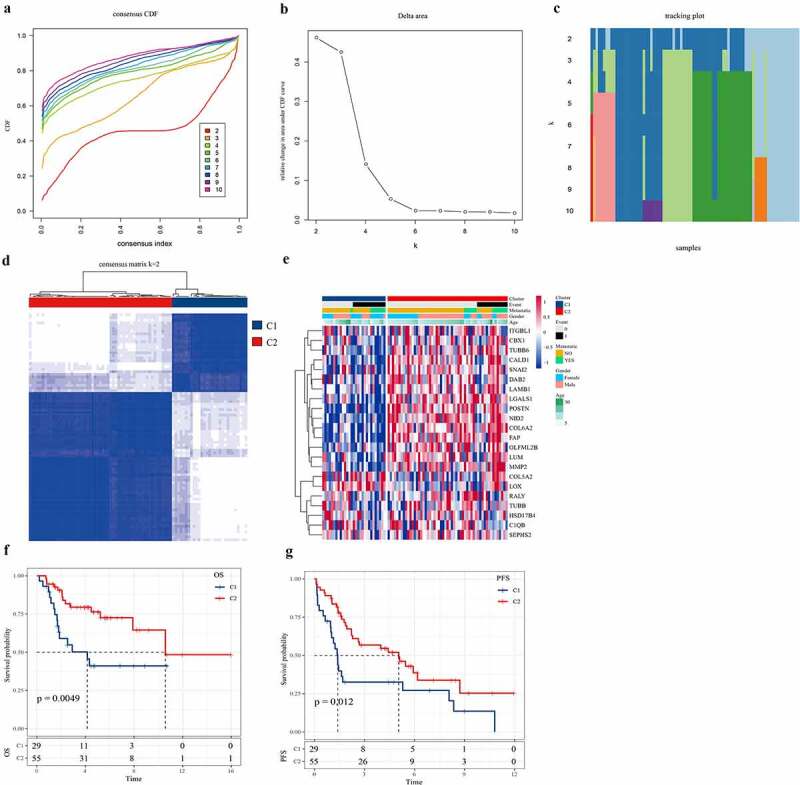
**Consensus clustering identified two molecular subtypes of** osteosarcoma **invasion.**

A total of 96 invasion genes were obtained using TARGET to filter the invasion-related genes obtained from CancerSEA (Table S1). Univariate Cox analysis showed that 22 out of the 96 invasion-related genes were significantly associated with the survival of osteosarcoma (*P* < 0.05) (Table S2). ConsensusClusterPlus was used to perform consensus clustering on the samples according to the 22 invasion-related genes. When the consensus index was 0.4–0.6 and k = 2, the empirical cumulative distribution function (CDF) curve was the flattest (Figure 2a, 2b). In addition, when k = 2, the consistency of the circular Manhattan (CM) was the highest, and the interference between subgroups was the lowest (Figure 2c, 2d). Therefore, the patients were divided into Cluster1 (C1) and Cluster2 (C2). The heatmap confirmed the expression of 22 invasion-related genes in C1 and C2 samples (Figure 2e). Moreover, survival analysis assessed the correlation between C1/C2, overall survival rate, and progression-free survival (PFS). We noted that there were significant differences in overall survival and PFS between the C2 subtype and C1 subtype, and that overall survival (OS) and PFS of subtype C2 were significantly longer than those of C1 (Figure 2f, 2g). Therefore, different molecular subtypes of osteosarcoma samples may have different clinical outcomes.

a: The CDF value of the consensus index. b: The relative change of the area under the CDF curve with k between 2 and 10. c: Tracking map of Kraft 2–10. d: The CM diagram shows the clustering at k = 2. e.g., 22 invasion-related gene clustering heat maps. f: Survival analysis assessed the association between C1 or C2 and overall survival. g: Survival analysis assessed the association between C1 and C2 and PFS.

### Immune score analysis of osteosarcoma samples

**Figure 3.**
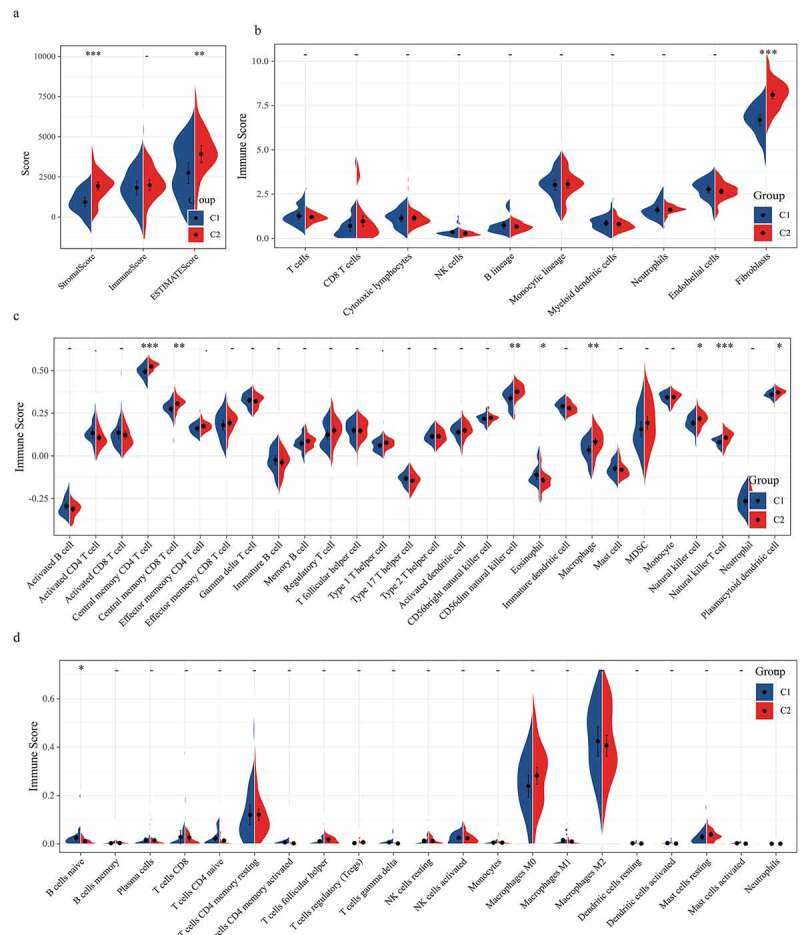
The relationship between two molecular subtypes and tumor immunity

We also analyzed the relationship between molecular subtypes and tumor immunity. Firstly, the differences of subtype C1 and C2 in stromal score, immune score, and ESTIMATE score, the stromal score, and ESTIMATE score of C2 were significantly higher than those of subtype C1 (Figure 3a). The scores of 10 immune cells between C1 and C2 subtypes were analyzed in MCPcounter, and a significant difference was found in fibroblasts immune scores between C1 and C2, with the immune scores of fibroblasts in C2 samples significantly higher than those in C1 samples (Figure 3b). According to the results of GSCA analysis, the immune scores of central memory CD4 T cell, central memory CD8 T cell, CD56dim natural killer cell, macrophage, natural killer cell, natural killer T cell, and plasmacytoid dendritic cell in subtype C2 samples were noticeably higher than those in subtype C1 samples, while the immune scores of Eosinophil in subtype C2 samples were significantly lower than those in subtype C1 samples (Figure 3c). As for the scores of 22 immune cells in the tissue samples calculated by CIBERSOTR, there was no significant difference in immune scores of 22 kinds of immune cells between C1 and C2 samples (Figure 3d).

a: The difference between C1 and C2 subtypes in stromal score, immune score, and ESTIMATE score. b: The immune scores of 10 kinds of immune cells in C1 and C2 subtypes were analyzed by MCP-counter. c: GSCA was used to compare the immune scores of 28 kinds of immune cells in different subtypes of samples. d: The scores of 22 kinds of immune cells in the tissue samples were calculated by CIBERSOTR.

### Identification of DEGs and functional enrichment analysis

**Figure 4.**
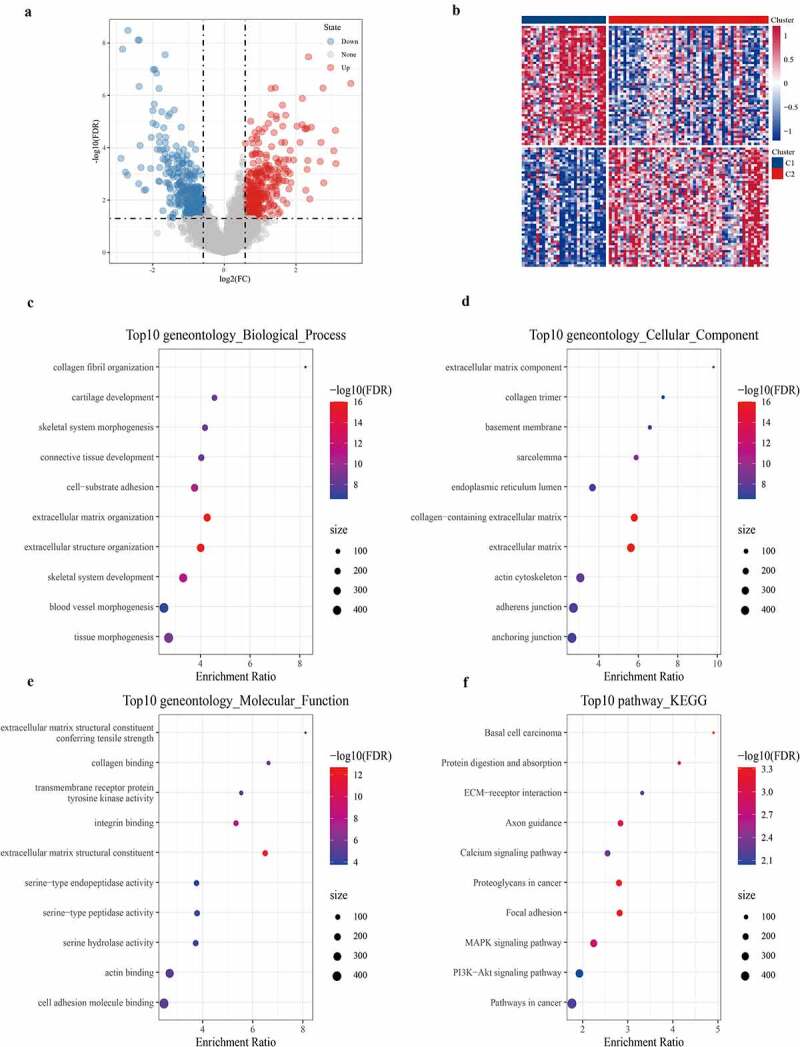
Identification and functional enrichment Analysis of DEGs

Differential analysis detected a total of 700 DEGs (359 differentially up-regulated genes and 341 differentially down-regulated genes) between C1 and C2 subtypes (Figure 4a, Table S3). The heat map of the top 100 genes with the most significant difference between the two subtypes was shown in Figure 4b. GO analysis on 700 DEGs demonstrated that the DGEs were significantly enriched into 327 biological processes (BP), 62 cellular components (CC), and 39 molecular functions (MF) GO terms (Table S4). According to the results of the GOBP analysis, 700 DEGs were significantly enriched in collagen fibril organization, cartilage development, skeletal system morphogenesis, and other processes (Figure 4c). GO CC analysis listed the 10 terms with the highest enrichment degree of DEGs (Figure 4d). From GO MF analysis, a significant correlation between DEGs and extracellular matrix structural constituents conferring tensile strength, collagen binding, and transmembrane receptor protein tyrosine kinase activity could be found (Figure 4e). This suggested that the 700 DEGs were closely related to bone development. Moreover, KEGG analysis demonstrated that 700 DEGs were significantly enriched into 22 pathways, such as basal cell carcinoma, protein digestion, and absorption, ECM–receptor interaction, calcium signaling pathway, and MAPK signaling pathway.

a: The volcano diagram of DEGs between C1 and C2 subtypes. b: Heat map of DEGs between C1 and C2 subtypes. c-e: The 10 BP terms, CC terms, and MF terms with the highest enrichment degree of DEGs. f: There were 10 pathways in which DEGs are significantly enriched.

### Construction and evaluation of prognostic signature

**Figure 5.**
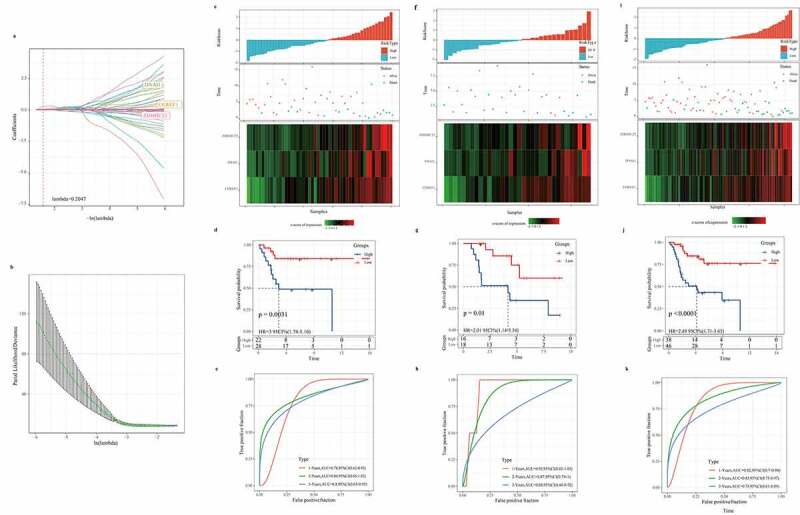
Construction and evaluation of prognostic signature

We analyzed the samples in the TARGET dataset. First, univariate Cox regression analysis detected 114 out of the 700 DEGs closely correlated with the overall survival of osteosarcoma patients (S5.csv). Next, the DEGs were analyzed using LASSO Cox regression, and a prognosis score system composed of three genes was developed (Figure 5a, 5b). Risk score = CGREF1*0.235+ DNAI1*0.457 + ZDHHC23*0.652. Each sample was graded according to this formula, and the standardized score was ranked from low to high. The survival status of patients was recorded, and the expression of three risk genes in the scoring model was analyzed, as presented in Figure 5c. Kaplan–Meier analysis showed that the survival rate of patients in the low-risk group was significantly higher than that in the high-risk group (Figure 5d). Based on the ROC analysis, the AUC of 1 year, 2 years, and 3 years were 0.78, 0.84, and 0.8, respectively (Figure 5e). The results revealed that the risk score effectively distinguished the survival time of patients in testing, TARGET validation set and entire TARGET data set (Figure 5f, 5i). Consistent with the training set, patients with high risk in the TARGET validation set and the whole TARGET data set were predicted to develop a (Figure 5g, 5j). The AUC of the predicted model for the training dataset at 1^st^ year, 2^nd^ year, and 3^rd^ year was 0.92, 0.87, 0.68, while 0.82, 0.85, and 0.75 for the whole TARGET data set, respectively (Figure 5h, 5k). The results indicated that our risk scoring system was highly accurate in predicting the prognosis of osteosarcoma.

a: The LASSO coefficient diagrams of 114 DEGs. b: LASSO regression with 5-time cross-validation. c, f, g: Training set, verification set, and total TARGET data set, the standardized risk scores were ranked from low to high (top), the survival status of patients was recorded (middle), and the expression of three risk genes in the scoring model was analyzed (bottom). d, g,j: Survival curves of patients in high-risk and low-risk groups in training set, verification set, and total TARGET data set. e, h, k: Survival curves of patients in high-risk and low-risk groups in training set, verification set, and total TARGET data set.

### Verification of the 3-gene signature in external datasets

**Figure 6.**
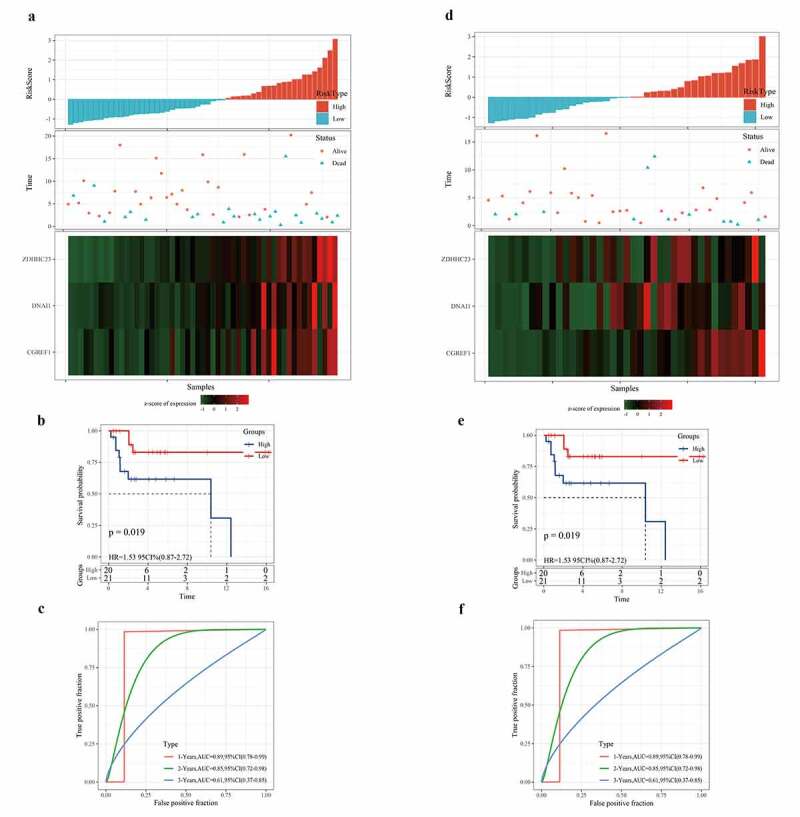
Verification of the robustness of the 3-gene signature in an external queue

To evaluate the versatility of the 3-gene signature, we also verified the prognostic performance of the 3-gene signature in independent cohorts of GSE21257 and GSE39058. Based on the risk scoring system, we obtained the risk score distribution, survival status, and three gene expression heat maps of the samples in GSE21257 and GSE39058 (Figure 6a, 6d). In both the GSE21257 cohort and the GSE39058 cohort, the risk type was significantly correlated with the overall survival rate of osteosarcoma patients (Figure 6b, 6e). From the ROC curve, AUC in the GSE21257 and the GSE39058 group were 0.82, 0.69, 0.75, and 0.89, 0.85, 0.61, respectively, for predicting 1-, 2-, and 3-year OS. Hence, the 3-gene signature was effective in predicting the prognosis of osteosarcoma (Figure 6c, 6f). Therefore, the above results show that the 3-gene signature can be used to predict the prognosis of patients with osteosarcoma in other independent cohorts.

a, d: Risk score distribution of samples in GSE21257 and GSE39058 (top), survival status (middle), and expression heat map of three genes (bottom). b, e: OS rate of patients with high or low risk. c, f: The ROC curve of 1-year, 2-year, and 3-year OS rates of osteosarcoma patients in GSE21257 and GSE39058.

### Independence of the 3-gene signature in prognosis prediction from clinicopathological factors

**Figure 7.**
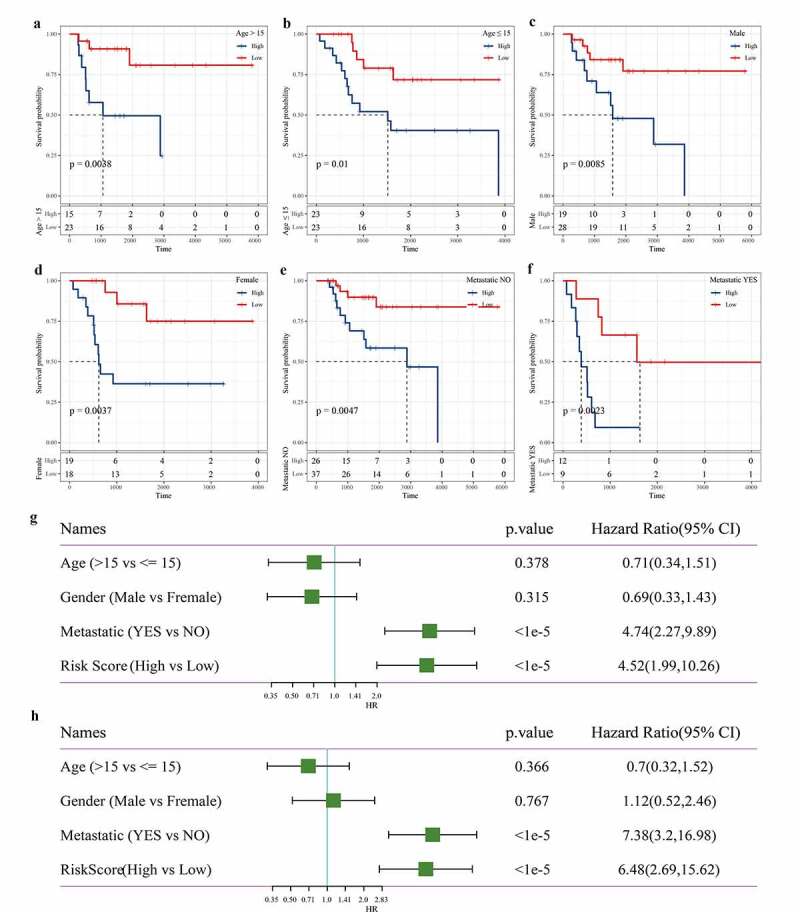
Independence of the 3-gene signature in prognosis prediction from clinicopathological factors

We also evaluated the applicability of the gene signature in predicting the prognosis of osteosarcoma based on age, sex, and metastasis. Survival analysis showed that regardless of age, gender difference, and metastasis, the survival rate of osteosarcoma patients in the low-risk group was significantly higher than that in the high-risk group (Figure 7a-7f). To assess the independence of 3-gene signature, we carried out univariate and multivariate Cox regression analysis. Univariate Cox analysis showed that metastasis and risk scores were closely correlated with the survival of osteosarcoma patients (Figure 7g). Multivariate Cox analysis demonstrated that metastasis and risk score was independent prognostic factors of osteosarcoma (Figure 7h). Therefore, the 3-gene signature was applicable and independent in predicting the prognosis of osteosarcoma.

a: The Kaplan–Meier curve of high and low-risk samples with age >15. b: The Kaplan–Meier curve of high and low-risk samples with age ≤ 15.c-d: The Kaplan–Meier curves of male and female patients with osteosarcoma. e: The Kaplan–Meier curve of osteosarcoma patients without metastasis. f: The Kaplan-Meier curve of osteosarcoma patients with metastasis. g: Univariate Cox analysis of the whole TARGET dataset sample. h: Multivariate Cox analysis of the whole TARGET dataset sample.

### The relationship between risk score and clinical characteristics

**Figure 8.**
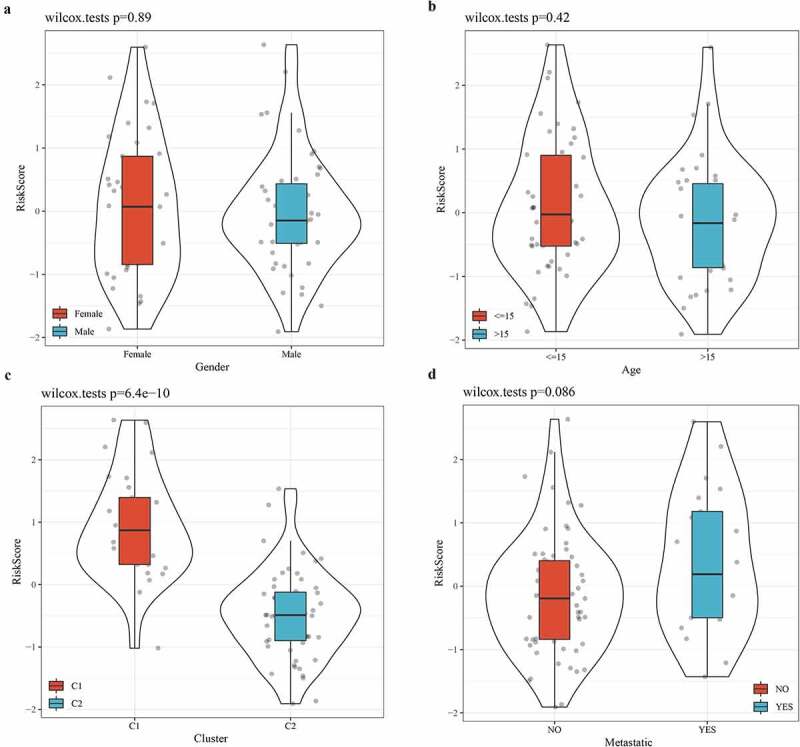
The relationship between risk score and clinical characteristics

To further verify the accuracy of the 3-gene signature in predicting the prognosis of osteosarcoma, the relationship between clinical features (sex, age, molecular subtype, and metastasis) and risk score was analyzed. From the violin picture, we noticed that the risk score was not significantly different between male and female groups, between age >15 and age <15 or between metastatic and non-metastatic groups (Figure 8a, 8b, 8d). However, there was a significant difference in risk score between subtype C1 and subtype C2, and C1 patients have a higher risk score, which could explain their worse prognosis (Figure 8c).

Violin chart, which was used to describe the relationship between risk score and gender (a), age (b), cluster (c), or metastasis (d).

### Construction of nomogram based on risk score and metastasis

**Figure 9.**
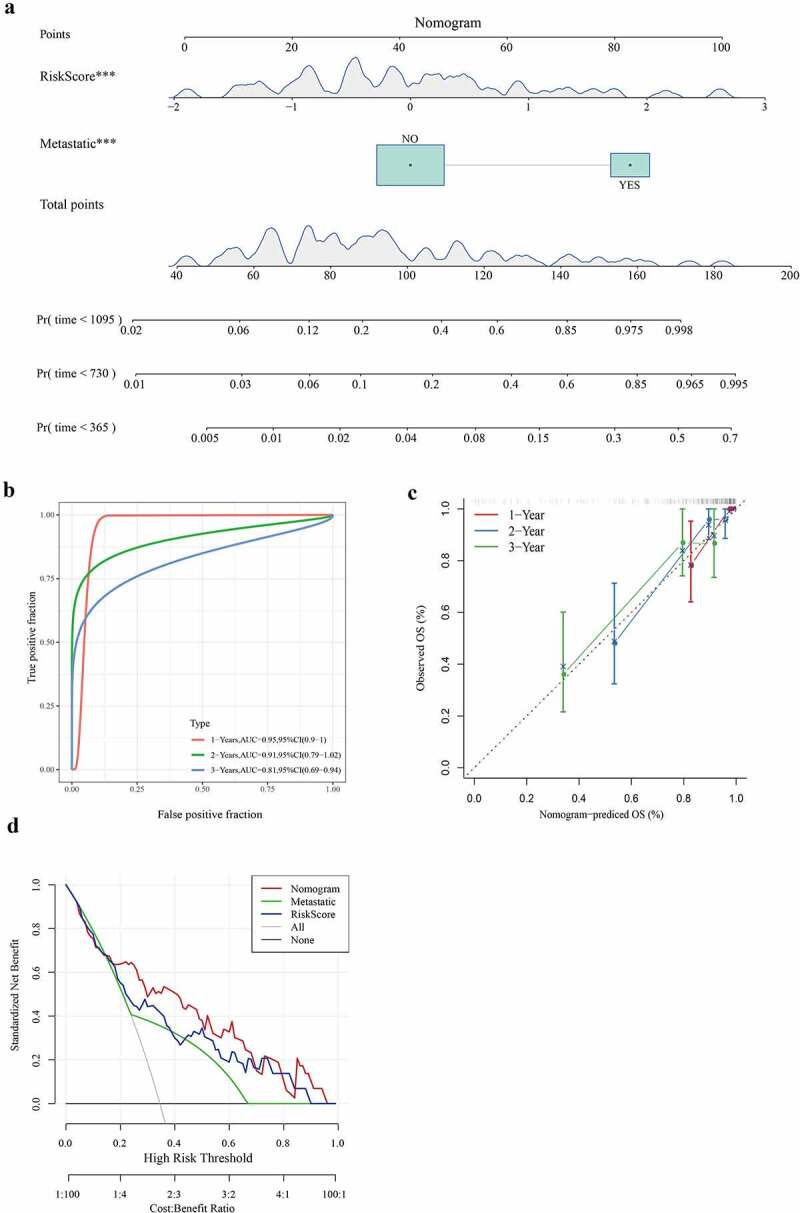
Construction of nomogram based on risk score and metastasis

We combined two independent prognostic factors (metastasis and risk score) to construct a nomogram for predicting 1-year, 2-year, and 3-year survival of patients with osteosarcoma. From the nomogram, we found that the risk score had the greatest influence on the prediction of survival (Figure 9a). The ROC curve showed that 1-year, 2-year, and 3-year AUC of the nomogram were higher than 0.8 (Figure 9b). The relationship between the predicted 1-year, 2-year, 3-year overall survival and the actual survival was evaluated using calibration chart, and it was found that the potential survival rate of osteosarcoma predicted by the nomogram was close to the actual survival rate (Figure 9c). From the DCA chart, the combination of the 3-gene signature and metastasis showed a certain net benefit in predicting the survival of osteosarcoma (Figure 9d). Therefore, the nomogram was verified to have a high prediction performance.

a: Nomogram for predicting 1-, 2-, and 3-year survival b: Calibration diagram of the Nomogram c: The DCA chart that evaluated net income.

### Comparison between the 3-gene signature and other known prognostic signatures

**Figure 10.**
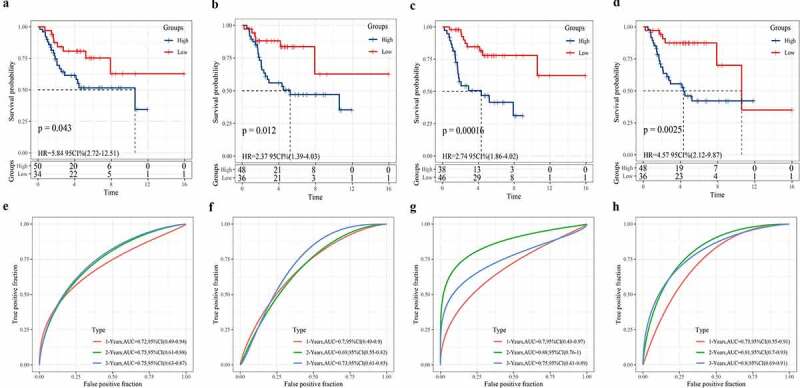
Comparison between the 3-gene signature and other known prognostic signatures

The 3-gene signature developed in this study was compared with the other four existing prognostic signatures. Based on the corresponding risk formula in these four models, the risk score of each osteosarcoma sample was calculated using TARGET. After standardization, the risk score was divided into high- and low-risk groups, with 0 as the critical value. Survival analysis showed that the survival rates of high-risk patients calculated according to the four risk score formulas were significantly lower than those of low-risk groups (Figure 10a-10d). The AUC of 1-year, 2-year, and 3-year was determined by ROC analysis. After comparing the AUC, we found that compared with the 19-gene signature (1 year: AUC = 0.72) (Figure 10e), the 8-gene signature (1 year: AUC = 0.7) (Figure 10f), the 6-gene signature (1 year: AUC = 0.7) (Figure 10g), and the 7-gene signature (1 year: AUC = 0.73) (Figure 10h), our 3-gene signature (1 year: AUC = 0.82) was more accurate in predicting the 1-year survival of OS. In addition, the AUC (AUC = 0.75) of 3-gene signature in predicting 2-year (AUC = 0.85) and 3-year survival of osteosarcoma was higher than or equal to 0.75 (Figure 5k). Compared with the other four signatures, our signature showed the least prognostic variables. Therefore, the 3-gene signature was an effective and accurate predictor of osteosarcoma prognosis.

a: The Kaplan Meier curves of patients in the high/low-risk group based on a 19-gene signature. b: ROC analysis of the 19-gene signature to estimate AUC values of survival. c: The Kaplan Meier curves of patients in the high/low-risk group based on an 8-gene signature. d: ROC analysis of the 8-gene signature to estimate the AUC values of survival. e: The Kaplan Meier curves of patients in the high/low-risk group are based on a 6-gene signature. f: ROC curve evaluated 1, 2, and 3-year prediction efficiency of the 6-gene signature. g: Kaplan–Meier curves of the high and low-risk groups according to a 7-gene signature. h: ROC curve evaluated 1, 2, and 3-year prediction efficiency of the 7-gene signature.

## Discussion

With the characterization of an increasing number of polygene prognostic models, studies on the prognosis of cancer based on specific tumor biological function-related genes gradually emerged. Some researchers have explored the prognostic potential of immune-related genes (IRGPs) in osteosarcoma, constructed an IRGP signature, and proved that the signature can accurately predict the overall survival of patients with osteosarcoma [23]. Naiqiang Zhu *et al.* developed a 7-gene signature related to the energy metabolism of osteosarcoma to predict the outcome of osteosarcoma [24]. Yucheng Fu *et al*. established a signature composed of two genes after analyzing hypoxia-related genes and speculated that it can be used as a biomarker for the prognosis of osteosarcoma [25]. In this study, we obtained 97 invasion-related genes from CancerSEA and 22 invasion-related genes related to osteosarcoma survival by univariate Cox analysis. It is reported that the characterization of specific molecular subtypes can facilitate clinical decision-making and the design of individualized treatment [26]. Therefore, we determined C1 and C2 osteosarcoma molecular subtypes by consensus clustering of invasion-related genes.

Immune cell group, which plays an important role in tumor development [27], has dual functions in tumor control and monitoring [28]. Here, we analyzed the immune cell scores of different subtypes in different platforms, and found significant differences in immune scores of fibroblasts, central memory CD4 T cell, central memory CD8 T cell, CD56 dim natural killer cell, macrophage, natural killer cell, natural killer T cell, plasmacytoid dendritic cell, and eosinophil between C1 and C2 subtypes.

A total of 700 DEGs between the C1 and C2 subtypes were identified, and functional enrichment analysis showed that they were closely related to bone development. Through univariate Cox analysis and LASSO Cox regression analysis, we constructed a signature based on three invasive genes. CGREF1, a protein secreted in the classical secretory pathway from endoplasmic reticulum to Golgi, has been found to play an important role in regulating the transcriptional activity of AP-1 and the proliferation of human colon cancer cells [29], but the role of CGREF1 in other cancers is unclear. The mutation of DNAI1 gene is a part of external power of ciliary organ, and is the second most important genetic cause of primary ciliary dyskinesia (PCD) [30]. Recently, it has been found that the risk model composed of CD180, MYC, PROSER2, and FATE1 has a great fitting effect on the overall lifetime of osteosarcoma [31]. Lijun Tian *et al.* reported that ZDHHC23 is palmitoyl transferase mainly located in Golgi apparatus and transGolgi network to control the palmitoylation of S0-S1 ring of BK channel to regulate surface transport [32]. At present, it is only known that ZDHHC23 can target glioma stem cells of different glioblastoma subsets and regulate the cellular plasticity of these subsets [33]. In this work, we were the first time to characterize the performance of the risk scoring system composed of three invasive gene on predicting the prognosis of osteosarcoma, and we found that the prognosis of patients with high risk of osteosarcoma was poor. The signature based on three invasive genes showed a high accuracy and independence in predicting the prognosis of osteosarcoma. In addition, the nomogram based on the signature and metastasis showed certain net benefits in predicting the survival of osteosarcoma, and may be a potential tool for predicting the prognosis of osteosarcoma patients.

Our research also has some limitations. First, the heterogeneity in osteosarcoma was high, which may affect the predictive performance of the signature. Second, there was limited clinical information and a lack of important pathological features such as tumor staging. Finally, no molecular experiments have been carried out to verify our predictions, but this will be addressed in future studies.

## Conclusion

In conclusion, our study identified two molecular subtypes of osteosarcoma based on invasion-related genes, developed a novel signature of three invasive genes, with a high accuracy, applicability, and independence in predicting survival of osteosarcoma patients. Our study may provide a new reference for osteosarcoma treatment.

## Supplementary Material

Supplemental Material

## Data Availability

The datasets used in this study were available at GSE21257 (https://www.ncbi.nlm.nih.gov/geo/query/acc.cgi?acc=GSE21257) and GSE39058 (https://www.ncbi.nlm.nih.gov/geo/query/acc.cgi).

## Research highlights:

- Two molecular subtypes of osteosarcoma were identified with invasion genes.
- C1 and C2 subtypes showed different tumor microenvironment states.
- We developed a gene signature based on three invasion genes.
- The signature had high accuracy and independence in the prognosis prediction.
